# Herbicides in vineyards reduce grapevine root mycorrhization and alter soil microorganisms and the nutrient composition in grapevine roots, leaves, xylem sap and grape juice

**DOI:** 10.1007/s11356-018-2422-3

**Published:** 2018-06-03

**Authors:** Johann G. Zaller, Clemens Cantelmo, Gabriel Dos Santos, Sandrina Muther, Edith Gruber, Paul Pallua, Karin Mandl, Barbara Friedrich, Ingrid Hofstetter, Bernhard Schmuckenschlager, Florian Faber

**Affiliations:** 1 Institute of Zoology, University of Natural Resources and Life Sciences Vienna (BOKU), Gregor Mendel Straße 33, A-1180 Vienna, Austria; 2 Federal College and Reseach Center of Viticulture and Pomology, Wiener Straße 74, A-3400 Klosterneuburg, Austria

**Keywords:** Agroecosystem, Arbuscular-mycorrhizal fungi, Belowground-aboveground interactions, Soil biota, Viticulture, Non-target effects, Weed control

## Abstract

Herbicides are increasingly applied in vineyards worldwide. However, not much is known on potential side effects on soil organisms or on the nutrition of grapevines (*Vitis vinifera*). In an experimental vineyard in Austria, we examined the impacts of three within-row herbicide treatments (active ingredients: flazasulfuron, glufosinate, glyphosate) and mechanical weeding on grapevine root mycorrhization; soil microorganisms; earthworms; and nutrient concentration in grapevine roots, leaves, xylem sap and grape juice. The three herbicides reduced grapevine root mycorrhization on average by 53% compared to mechanical weeding. Soil microorganisms (total colony-forming units, CFU) were significantly affected by herbicides with highest CFUs under glufosinate and lowest under glyphosate. Earthworms (surface casting activity, density, biomass, reproduction) or litter decomposition in soil were unaffected by herbicides. Herbicides altered nutrient composition in grapevine roots, leaves, grape juice and xylem sap that was collected 11 months after herbicide application. Xylem sap under herbicide treatments also contained on average 70% more bacteria than under mechanical weeding; however, due to high variability, this was not statistically significant. We conclude that interdisciplinary approaches should receive more attention when assessing ecological effects of herbicides in vineyard ecosystems.

**Electronic supplementary material:**

The online version of this article (10.1007/s11356-018-2422-3) contains supplementary material, which is available to authorized users.

## Introduction

With an increasing intensification of viticulture, chemical weed control within and between grapevine (*Vitis vinifera* L.) rows have been widely employed. Herbicides are used (i) to lessen competition for water and nutrients caused by weeds, (ii) to avoid trunk damage caused by mechanical weeding machinery, and (iii) to reduce working time spent in the vineyard (Keller 2015). As a result of their wide use, herbicide residues can be found in considerable amounts in ground and surface waters (Herrero-Hernández et al. 2017) as well as in wine (Ying and Williams 1999; Ying and Williams 2000). Among the most often used herbicides in vineyards are those based on the active ingredients glyphosate, glufosinate and flazasulfuron (Bauer et al. 2017). While effects of fungicides and/or insecticides on soil organisms in vineyards have been reported (Paoletti et al. 1998), not much is known on the impacts caused by herbicides (Stellin et al. 2017). While it can be expected that the mode-of-action of herbicides on non-target organisms in vineyards should be similar to those of arable fields, also different effects could be anticipated. First, mobile soil organisms such as earthworms might be less affected in vineyards as herbicides are often applied only underneath the grapevine rows leaving the inter-row area untreated. Second, vineyards are generally more intensively managed than arable fields including more frequent disturbance and pesticide applications that could interact with herbicides concurrently applied (van Hoesel et al. 2017). Third, as perennial crops, grapevines might be more susceptible to weed control via herbicides than annual crops because of a chronical herbicide-stress over the years.

In vineyards, as in many other agroecosystems, the role of earthworms in promoting soil fertility, aggregate formation and soil organic matter protection is important (Lavelle et al. 1997). Earthworms have also been considered as sensitive bioindicators for a sustainable vineyard management (Paoletti et al. 1998; Pérès et al. 2008). Glyphosate-herbicides used for weed control in vineyards have been linked to increased earthworm mortality especially for deep-burrowing earthworm species (Stellin et al. 2017). On the other hand, it was shown that mechanical weeding in vineyards through tillage is not necessarily detrimental to earthworms as it usually applies during times when earthworms are in deeper soil layers (Faber et al. 2017). Additionally, weeds growing in vineyard inter-rows can buffer negative tillage effects on earthworms and other soil biota (Buchholz et al. 2017). Earthworms have also been shown to affect root growth (Arnone and Zaller 2014) and their colonisation with symbiotic arbuscular mycorrhizal fungi (AMF; Zaller et al. 2011b; Trouvé et al. 2014).

Colonisation of grapevine roots with AMF has been shown to increase grapevine (i) growth and nutrition, (ii) tolerance to water stress and (iii) protection against diseases (Trouvelot et al. 2015). The role of other soil microorganisms such as yeasts, moulds or bacteria in vineyard soils are less well studied. However, findings indicate that besides climatic and pedological characteristics, soil microorganisms also alter grapevine physiology and are even responsible for a typical terroir of wines (Bokulich et al. 2014; Gilbert et al. 2014).

The aim of the current study was to examine non-target effects of chemical and mechanical weed control on soil biota and grapevine nutrition. Because earthworms (Gaupp-Berghausen et al. 2015), mycorrhizal fungi (Zaller et al. 2014) and also soil microorganisms (Kopčáková et al. 2015; Aristilde et al. 2017) have been shown to be affected by chemical herbicides, we hypothesised that herbicide-induced alterations would be evident through changes in plant as well as soil. It has been shown that herbicide use in vineyards affect physiological parameters in the non-target grapevine crop leading a decrease of the leaf dry weight percentage and soluble carbohydrate content with suggested consequences for the berry growth, free amino acid content and an accumulation of ammonium (Saladin et al. 2003). A better understanding of side effects of different weed control methods within the vineyard ecosystem would help to develop more ecologically sound viticultural management practices (Likar et al. 2017).

## Materials and methods

### Study site

The study was conducted in a vineyard of the experimental winery Agneshof of the Federal College and Reseach Center of Viticulture and Pomology in Klosterneuburg, near Vienna, Austria (48° 17′ 39.03″ N, 16° 19′ 26.18″ E, 190 m a.s.l.). The vineyard was established in 2012 with the white grape variety Gewürztraminer using trellis (grapevine within-row distance, 1.0 m; row distance, 2.8 m). Soils at the study site developed from alluvial soils of sandy, brown primary material and rounded pebble stones; chiselled Flysch marl stemmed from culluvial processes. The vineyard is south-facing, slightly inclined, and the inter-rows were cultivated according to the Austrian soil erosion prevention programme allowing only tillage of every second inter-row, while leaving the other rows uncultivated and vegetated (ÖPUL 2007). Fertilisers and fungicides were applied evenly across the vineyard following good viticultural practice.

The field study was conducted between March and July 2016; additionally, earthworms were extracted in November 2016 and cultivated in the laboratory until July 2017. Samples for xylem sap were collected in April 2017. Precipitation during the field investigations amounted to 45.8 mm in April, 150 mm in May and 85 mm in June at a mean air temperature of 10.7, 15.0 and 19.4 °C, respectively. Long-term (years 1970–2000) mean annual air temperature at this location is 10 °C, mean annual precipitation 620 mm (Zentralanstalt für Meteorologie und Geodynamik, Vienna).

### Herbicide treatments

Effects of three different broadband herbicide applications and one mechanical weeding within grapevine rows were tested. Each treatment was applied along eight grapevines and was applied in two spatially distinct inter-rows. There was a distance of 5 m between different treatments in the same row, to avoid cross-contamination. Each treatment row was neighboured by two rows with mechanical weeding in order to avoid potential cross-contamination of different treatments by herbicide drift.

Herbicide treatments were employed according to recommendations of the manufacturer and/or good viticultural practice (http://pmg.ages.at/; Table 1). Herbicide one contained the active ingredient flazasulfuron that inhibits the amino acid synthesis, cell division and ultimately plant growth (Basta 150 SL, Bayer CropScience Austria, Vienna, Austria). Herbicide two contained the active ingredient glufosinate that inhibits glutamine production, halting photosynthesis and resulting in plant death (Katana, ISK Biosciences Europe, Brussels, Belgium). Herbicide three contained the active ingredient glyphosate as potassium salt and is a non-selective systemic broadband herbicide inhibiting the glutamine synthetase in plants (Roundup PowerFlex, Monsanto Agrar Deutschland, Düsseldorf, Germany). The chemical herbicides were sprayed by an experienced agro-technician of the research station using a backpack sprayer at windless condition; the treated strip within rows was about 50 cm wide and 9 m long for each herbicide. The control treatment consisted of mechanical weeding performed by hand using a weeding hoe. Vegetation within grapevine rows was about 20 cm tall when weed control measures were applied. The tested herbicides are frequently used in Austrian vineyards.

**Table 1:** Overview of within-row weed management treatments employed in the current experiment

Active ingredient/treatment	Product name	Conc. active ingredient	Dosage applied (all dates)	Application date in 2016
Mechanical weeding	n.a.	n.a.	n.a.	April 14
Flazasulfuron	Katana	250 g kg^−1^	200 g ha^−1^	April 7
Glufosinate	Basta 150 SL	200 g l^−1^	3.75 l ha^−1^	April 7 + June 7
Glyphosate	Roundup PowerFlex	200 g l^−1^	4.0 l ha^−1^	April 7

*n.a.* not applicable

### Sampling and measurements

In the study vineyards, we assessed effects of herbicide treatments on earthworms (activity, density, biomass, reproduction); on soil microorganisms (colony-forming units of bacteria, yeasts and molds); litter decomposition in soil; grapevine root mycorrhization; and nutrient concentrations in grapevine roots (N, P, K, Ca, Cu, Fe, Mg, Mn, Zn), leaves (N, P, K, Ca, Cu, Fe, Mg, Mn, Zn), xylem sap (P, K, Ca, Mn; bacteria and fungi) and grape juice (N, P, K, phenolics).

Earthworm activity in grapevine rows was assessed four times (April 5, June 11, June 18, June 23, 2016) by counting the surface casts on two marked 30 × 30-cm quadrats per treatment replicate. Number of casts were counted, collected, dried at 50 °C for 48 h and weighed. To assess earthworm density and biomass within treatment inter-rows, one soil cube (25 × 25 × 25 cm, length × width × depth) was excavated using a spade on June 25, 2016 in 20-cm distance from a grapevine in order. Soil cubes were carefully searched for earthworms; found earthworms were separated into juveniles and adults, counted, cleaned from attached soil and weighed. In November 2016, another earthworm sampling was performed by excavating the same soil cubes. All earthworms from these soil cubes were sorted out, transferred to plastic boxes (16 × 11 × 4.5, L × W × H), filled with 2-cm soil and cultivated in a laboratory under room temperature (10 °C, 24-h darkness). The development of these earthworms was monitored over 33 weeks. On average, every 4 weeks, we recorded the number of individuals, produced cocoons and emerging hatchlings. Earthworm species were identified using the key of Christian and Zicsi (1999).

Soil microorganisms were determined on two bulk soil samples (5 × 5 × 10 cm, L × W × H) per treatment replicate taken in 25 June at a depth between 10 and 20 cm within two treated grapevines. Colony-forming units (CFU) of bacteria, yeasts and molds were determined on 1 g of each soil sample that was diluted according to the serial dilution method (Ben-David and Davidson 2014). Therefore, a sample with an unknown number of CFUs was diluted in a series of dilutions with a certain dilution factor. A series of nine dilutions were made with a factor of 10. One gram of soil was diluted with 9 ml of a 0.9% NaCl solution. Hundred microliters of each dilution was then plated on three different agar plates. The substrates used were Malt Extract Agar (Weidenbörner 1998), Wallerstein nutrient agar (17222-500G, Merck, Darmstadt, Germany) and Tryptone Soy Agar (CP 70.1, Carl Roth, Karlsruhe, Germany). These different substrates simplified the differentiation of the three analyzed microorganism groups: bacteria, yeasts and molds. After 6 days of incubation at room temperature, the CFUs were counted. The general ranges in common acceptance for countable numbers of colonies on a plate are 30–300 and 25–250 (Sutton 2011). Bacteria, yeasts and molds were then visually differentiated; the molds were classified visually, if fruiting bodies were detected.

Litter decomposition in the soil was determined using the Tea Bag Index (TBI; Keuskamp et al. 2013). Therefore, five plastic tea bags containing green tea (Lipton Unilever, USA: EAN 87 22700 05552 5) and five teabags with rooibos tea (Lipton: EAN 87 22700 18843 8) were dried, weighed and buried at 10-cm depth per treatment replicate. Tea bags were removed 80 days after insertion, dried at 70 °C for 48 h and weighed again. Afterwards, the decomposition rate (*k*) and stabilization factor (*S*) were calculated; the recommended calculated hydrolysable fractions (0.842 g g^−1^ for green tea; 0.552 g g^−1^ for rooibos tea) were used (www.teatime4science.org/method/stepwise-protocol/). During decomposition, parts of the labile compounds stabilise and become recalcitrant. This stabilization depends on environmental factors and results in a deviation of the actual decomposed fraction (i.e. limit value) from the hydrolysable (i.e. chemically labile) fraction. Stabilisation factor *S* is this deviation and interpreted as the inhibiting effect of environmental conditions on the decomposition of the labile fraction.

Roots were sampled directly from the grapevines growing in the treated rows. Therefore, on one side of the grapevine, the root system was carefully excavated, and about 15 cm of second-grade roots was cut off directly from the rootstock. To determine mycorrhization, roots were washed free of attached soil, cut into small pieces of about 2 mm, bleached in 10% potassium hydroxide solution for 4 min and stained using a 5% vinegar-ink solution for 1 min, cleaned with tap water and stored in 50% ethanol (Vierheilig et al. 1998). The rate of mycorrhization was determined under a binocular (× 40 magnification) using the gridline intersect method by counting at least 100 intersection points (Giovanetti and Mosse 1980). This measurement of mycorrhization was performed twice on randomised root samples. Afterwards, root samples were dried at 50 °C for 48 h and weighed. For nutrient analyses, root samples were taken on 22 June 2016 and on 16 August 2016. Therefore, roots of three grapevines per treatment up to a maximum length of 30 cm were excavated to a depth of about 15 cm. Roots were carefully cleaned from attached soil, cut into small pieces and stored in paper bags at room temperature until further analysis.

Grapevine leaf nutrient concentrations were determined on leaves sampled at the same dates as root sampling. Therefore, 20 leaves were taken per treatment replicate from the grape zone, opposite the first grape. Care was taken that only healthy and undamaged leaves were collected. Later, we removed the leaf stalks and placed the leaf blades in paper bags and dried at 60 °C for 24 h.

Grapevine xylem sap was collected in spring 2017 (11 months after herbicide applications) before grapevine bud break. Therefore, we first disinfected a fruiting rod with alcohol (90%) and induced sap bleeding by recutting the end of one cane of one grapevine per treatment replicate. Sap was collected between 24 and 29 March 2017 using a 0.5-L plastic bottle attached to the grapevine stem and wrapped with aluminium foil to prevent the influence by sunlight (overheating, influencing nitrogen substances). From this sap sample, a part was saved for nutrient analysis (see below). Another part sap was transferred into two 50-ml centrifuge tubes and brought into the laboratory. In the lab, we performed pure cultures from each sap sample in order to sequence their DNA. Molds, which did not show any fruiting bodies or which could not clearly be identified, were chosen. This allowed having a more accurate representation of the mold diversity, since visual differentiation sometimes was not effective. A small part of the chosen colony was taken with a scalpel and plated on another sterile agar plate with the same nutrient on which the colony was previously found. After 6 days of incubation and only, if the colony appeared to be pure, a part of this colony was again taken with a scalpel and then put into a liquid malt extract agar medium for molds. After another 6 days, when the colony was clearly identifiable, DNA extraction was made. The DNA extraction was performed according to the Master Pure™ DNA-Purification Kit from Epicentre. For fungi, we used the primers ITS1 (TCCGTAGGTGAACCTGCGG) and ITS4 (TCCTCCGCTTATTGATATGC) surrounding the 5.8S-coding sequence (White et al. 1990; Gardes and Bruns 1993), and for bacteria, we used the AC1 and AC3 primers (Poblet et al. 2000) to perform the PCR sequences surrounding the 16S-coding sequence (Woese and Fox 1977). PCR products were performed on gel-electrophoresis. The recognizable DNA templates were cut out from the gel. DNA purification was performed with the Wizard SV Gel and PCR Clean-Up System Kit and according to its instructions. The reactions were performed in a Mastercycler (Eppendorf, The Netherlands). For the amplification of the fungi primers ITS1 and ITS4, the following protocol was used: 95 °C 5 min^−1^ − (55 °C min − 72 °C 1.5 min) × 35 − 72 °C 5 min. After the amplifications, the DNA samples were stored at 4 °C. For the amplification of the bacteria primers AC1 and AC3, the following protocol was used: 94 °C 5 min − (94 °C 1.0 min − 62 °C 2.0 min − 72 °C 2.0 min) × 35 − 72 °C 10 min. After the amplification, the DNA samples were stored at 4 °C. For the PCR reaction mixture, we used 1.5 μl Primer forward, 1.5 μl Primer reversed, 17 μl H_2_0, 25 μl PCR Master Mix (Promega, USA) and 5 μl DNA. The purified DNA was sent to the Eurofins laboratories (Eurofins Scientific, Berlin, Germany) to be sequenced and then compared through the Basic Local Alignment Search Tool (BLAST; https://blast.ncbi.nlm.nih.gov/Blast.cgi). See supplementary Table S1 for a condensed BLAST output.

Grape juice was sampled on September 7, 2016 by randomly collecting 50 fully ripe grape berries from different grapevines per treatment. Samples were subsequently processed with a juice extractor and analysed. Concentrations of N in xylem sap and grape juice were analysed after a sulfuric acid digest with subsequent determination after Kjeldahl. Analysis of P was analysed spectrophotometrically using a modified molybdenum blue method (Barna and Grill 1980). All other elements were analysed with a mass spectrometer using a Multiwave 3000 and inductively coupled plasma device (ICP Spectrometer, iCAP 6000 Series; Thermo Fischer Scientific, Cambridge, UK). Phenolics in grape juice were analysed using high-performance liquid chromatography (HPLC; Agilent Technology 1200, Santa Clara, CA, USA) using the ZORBAX SB-C18 column (15 × 2.1 mm; 1.8 μm; Vrhovsek et al. 1997).

### Statistical analysis

First, parameters were tested for normality and variance homogeneity using P-P plots and Levene tests. Second, effects of herbicide treatment (four levels: three herbicide treatments and one mechanical weeding) were tested on cumulative earthworm activity, earthworm numbers and biomass, litter decomposition rate, litter stabilisation factor, grapevine root mycorrhization rate, soil microbial colony-forming units and proportion of taxa to microbial community; nutrients in xylem sap were tested using one-way Analysis of Variance (ANOVA). When treatment effects were significant, mean comparisons between treatments were analysed using post hoc Tukey tests. A MANOVA on P and K concentrations in different grapevine parts was performed to evaluate if there is a shift in these nutrients between grapevine parts under different weed control regimes. To investigate relationships between earthworms or mycorrhizal colonisation and nutrient contents, Pearson correlations were performed. All data were analysed using the software IBM SPSS Statistics (vers. 24, IBM Incorporation, Armonk, NY, USA). Values given within the text are means ± one standard deviation.

## Results

**Fig. 1:**
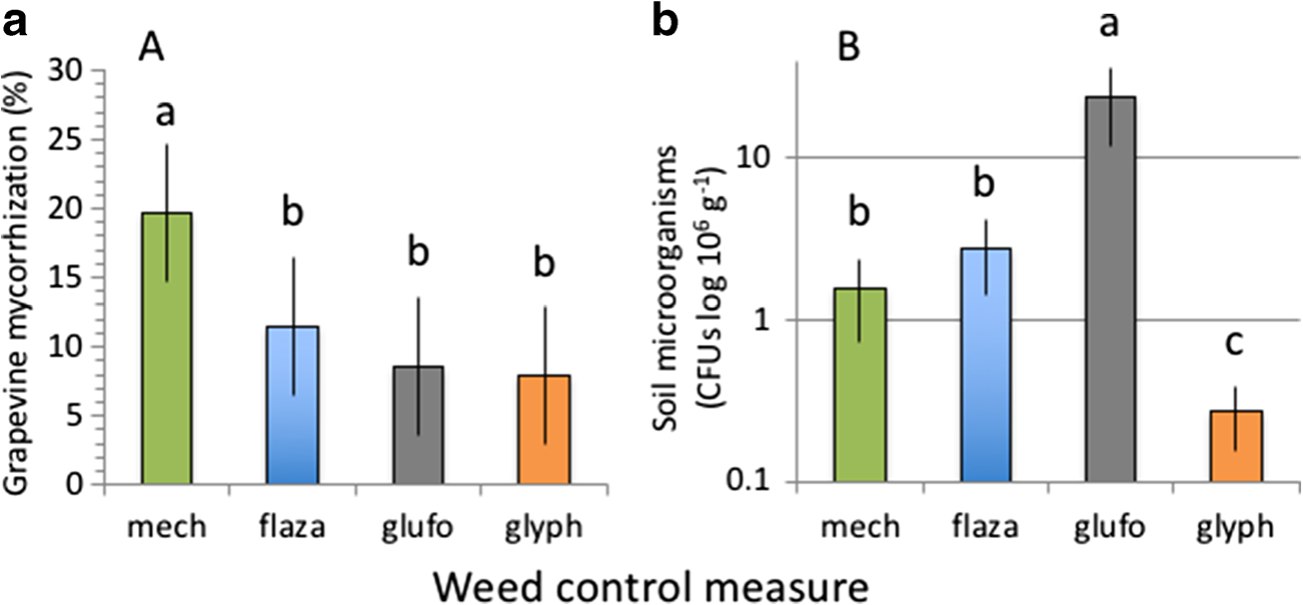
Grapevine root mycorrhization (**a**) and soil microorganisms (**b**) in response to mechanical (mech) and chemical flazasulfuron (flaza), glufosinate (glufo) and glyphosate (glyph) weed control measures. Different letters above bars denote significant differences between treatments. Means ± SD, *n* = 5

Grapevine mycorrhization was significantly affected by weed control treatments (Fig. 1a, Table 2). The highest mycorrhization (19.7 ± 4.0%) was observed under mechanical weed control; mycorrhization was significantly lower under herbicide applications but did not vary between different herbicides (averaged across herbicides 9.3 ± 3.3%; Fig. 1a).

**Table 2:** ANOVA results for the effects of mechanical weeding and three herbicides (active ingredients: glufosinate, flazasulfuron, glyphosate) on grapevine root mycorrhization, soil microorganisms, earthworms (EW), litter decomposition and xylem sap bacteria. *F* statistics and *P* values; significant effects in bold

Parameter	Weed control measure	
	*F*	*P*
Grapevine mycorrhization (%)	9.846	**0.001**
Soil total colony-forming units (CFUs)	3.305	**0.022**
Soil yeast abundance (CFUs)	1.152	0.330
Soil molds (CFUs)	1.008	0.391
Soil bacteria (CFUs)	2.321	0.077
EW biomass (g m^−2^)	0.875	0.481
EW density (no. of m^−2^)	1.178	0.359
EW individual biomass (g EW^−1^)	1.030	0.414
EW activity (total no. of surface cast production)	0.600	0.627
EW activity (no. of casts per EW)	1.066	0.423
EW cocoon production (no. of cocoons)	0.567	0.643
EW reproduction (no. of hatchling cocoon^−1^)	0.757	0.531
Litter decomposition rate (*k*)	0.333	0.802
Litter stabilization factor (*S*)	0.176	0.912
Xylem sap bacteria number of taxa	1.667	0.227
Xylem sap bacteria abundance (total CFUs)	2.388	0.120

Soil microorganisms (colony-forming units, CFU) were significantly affected by the employed weed control treatments (Fig. 1b, Table 2). Significantly, more CFUs were found under glufosinate (24.2 ± 12.3 10^6^ g^−1^) than under mechanical weeding or under flazasulfuron (averaged 2.2 ± 1.1 10^6^ g^−1^); least CFUs were observed under glyphosate (0.3 ± 0.1 10^6^ g^−1^; Fig. 1b).

**Fig. 2:**
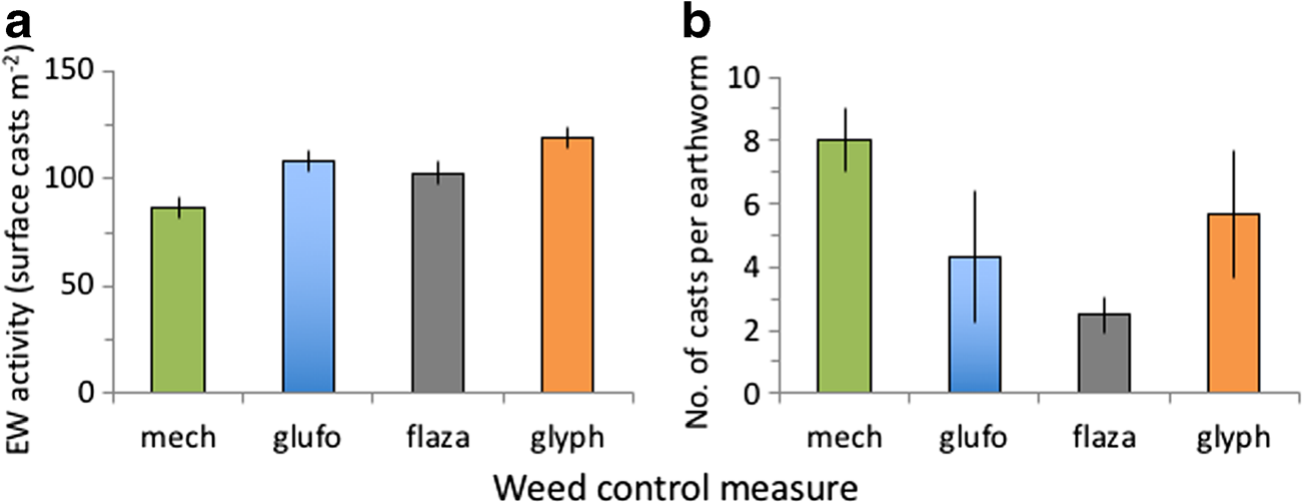
Earthworm activity measured in surface casts (**a**) or number of casts produced per earthworm (**b**) in response to mechanical (mech) and chemical flazasulfuron (flaza), glufosinate (glufo) and glyphosate (glyph) weed control measures. No significant differences between treatments were observed. Means ± SD, *n* = 5

Earthworm biomass, density, surface casting activity (Fig. 2) and reproduction were unaffected by weed control measures (Table 2, Table 3).

**Table 3:** Earthworm parameters and litter decomposition in vineyard inter-rows with mechanical (mech) and three chemical weed control measures (flaza…flazasulfuron, glufo…glufosinate, glyh…glyphosate). Means ± SD. No significant differences between weed control measures were observed for these parameters

Parameter	Weed control measure			
	mech	flaza	glufo	glyph
Earthworm biomass (g m^−2^)	0.6 ± 1.1	12.5 ± 10.0	12.5 ± 5.3	8.9 ± 15.6
Earthworm density (no. of m^−2^)	2.8 ± 5.6	30.6 ± 30.6	47.2 ± 22.9	41.7 ± 61.8
EW indiv. Biomass (g EW^−1^)	0.6 ± 1.1	4.4 ± 3.7	5.2 ± 8.1	1.1 ± 1.3
EW cocoon production (no.)	2.3 ± 2.1	7.3 ± 12.2	4.5 ± 7.6	1.2 ± 1.3
EW hatchling (no. of cocoon^−1^)	2.5 ± 2.1	6.3 ± 12.2	5.8 ± 7.6	1.0 ± 1.3
Litter decomposition rate (*k* 10^−2^)	2.05 ± 1.16	1.94 ± 1.12	1.57 ± 0.77	1.86 ± 0.86
Litter stabilization index (*S* 10^−1^)	4.44 ± 0.49	4.41 ± 0.43	4.48 ± 0.54	4.31 ± 0.55

Litter decomposition rate (averaged across treatments 18.6 ± 3.3 10^−3^) and stabilization index (averaged across treatments 440.9 ± 16.8 10^−3^) were unaffected by weed control measures (Table 2, Table 3).

**Fig. 3:**
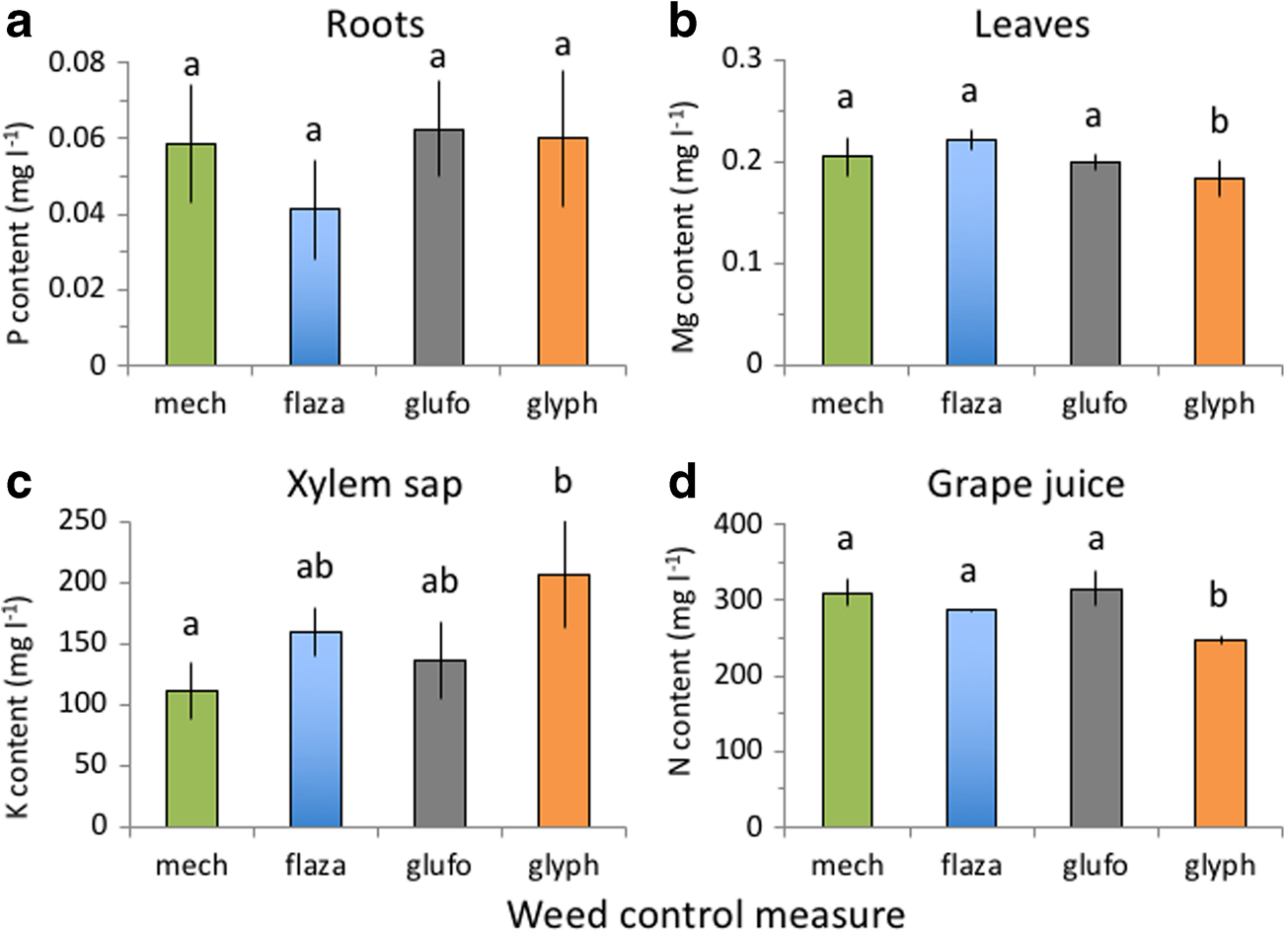
Nutrient concentrations in grapevine roots (**a**), leaves (**b**), xylem sap (**c**) and grape juice (**d**) in response to mechanical (mech) and chemical flazasulfuron (flaza), glufosinate (glufo) and glyphosate (glyph) weed control measures. Different letters above bars denote significant differences between treatments. Means ± SD, *n* = 5

Nutrient contents in different parts of the grapevines were differently affected by weed control. Root P content was marginally (Fig. 3a), grapevine leaf Mg significantly and P and K marginally (Fig. 3b), xylem sap K significantly (Fig. 3c) and grape juice N content also significantly (Fig. 3) affected by weed control measures (Table 4). MANOVA analyses (Pillai lambda) for P and K concentrations in different grapevine parts showed significant effects of herbicide treatment (*F* = 4.034, *P* = 0.002), grapevine part considered (*F* = 210.560, *P* < 0.001) and an interaction between herbicide treatment and grapevine part (*F* = 2.753, P 0.001).

**Table 4:** ANOVA results for the effects of one mechanical and three chemical weed control measure employed within-row in a vineyard on nutrient contents in grapevine roots, leaves, xylem sap and grape juice. All units are in milligrams per liter. *F* statistics and *P* values; significant effects in bold

Parameter	Weed control	
	*F*	*P*
Roots		
Nitrogen	0.565	0.650
Phosphorous	3.261	0.081
Potassium	0.161	0.921
Calcium	1.546	0.254
Copper	1.066	0.400
Iron	0.135	0.937
Magnesium	0.503	0.688
Manganese	0.244	0.864
Zinc	1.209	0.348
Leaves		
Nitrogen	0.071	0.975
Phosphorous	2.998	0.073
Potassium	2.896	0.079
Calcium	0.065	0.977
Copper	0.008	0.999
Iron	0.300	0.825
Magnesium	4.633	**0.023**
Manganese	1.447	0.278
Zinc	1.139	0.378
Xylem sap		
Phosphorous	0.858	0.489
Potassium	4.969	**0.037**
Calcium	1.143	0.371
Manganese	0.508	0.686
Grape juice		
Nitrogen	9.241	**0.029**
Phosphorous	0.127	0.939
Potassium	2.068	0.247
Phenolics	1.000	0.479

Across treatments, xylem sap K concentration was significantly negatively correlated with grapevine mycorrhization (*r* = − 0.702, *P* = 0.016), sap P concentration was significantly negatively correlated with earthworm density (adult earthworms only, *r* = − 0.599; *P* = 0.014) and significantly negatively correlated with earthworm biomass (*r* = − 0.501, *P* = 0.048). Also, there was a marginal significant trend negative relationship between earthworm density and grapevine mycorrhization (*r* = − 0.482, *P* = 0.059).

**Fig. 4:**
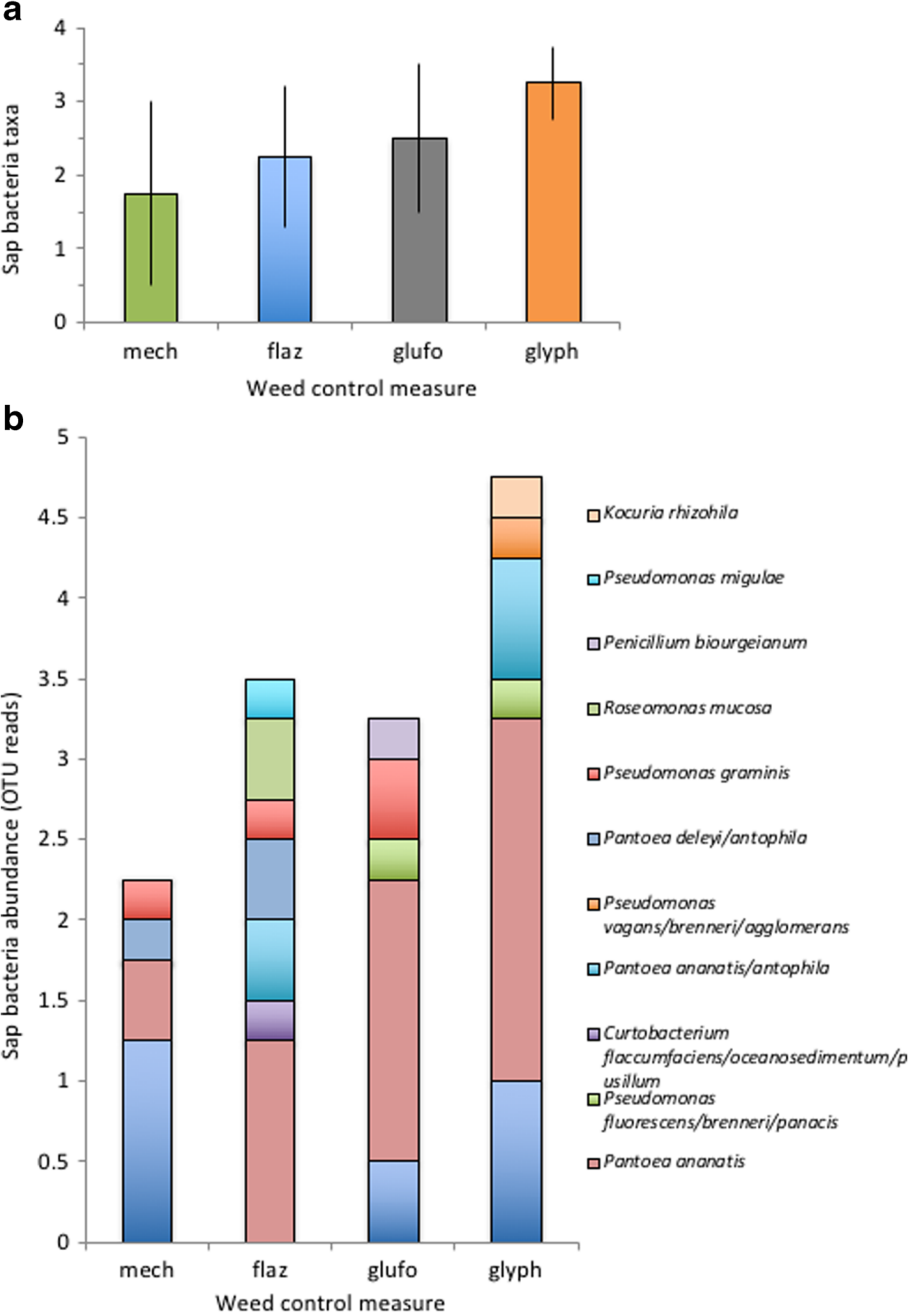
Xylem sap bacteria taxa (**a**) and total abundance (**b**) in response to mechanical (mech) and chemical flazasulfuron (flaza), glufosinate (glufo) and glyphosate (glyph) weed control measures. Abundance is defined as the number of bacterial isolates of a given OTU. Means ± SD, *n* = 5

Mean number of bacteria taxa in the xylem sap was similar between weed control treatments (averaged across treatments 2.4 ± 0.9, Fig. 4a). Total abundance of sap bacteria was higher under chemical weed control (averaged 3.8 ± 1.2 reads) than under mechanical control (2.2 ± 1.7 reads); however, this was not statistically significant (Fig. 4b, Table 2).

## Discussion

This is among the first field studies showing that herbicides commonly used in vineyards can affect various non-target parameters and processes within the vineyard ecosystem. Among the most prominent herbicide-induced effects was a 53% decrease in grapevine mycorrhization. This detrimental effect was unrelated to the three different active ingredients investigated, suggesting that either non-target effects on grapevine physiology or adjuvants mixed into the herbicide formulations might be responsible for this effect. Studies have shown that pesticide adjuvants can have more toxic effects on non-target organisms than the actual active ingredient (Mullin et al. 2016; Mesnage and Antoniou 2018). However, unfortunately, these adjuvants are usually considered as company secrets and are very less like investigated. Mycorrhization has been shown to be affected by glyphosate-based herbicides in herbal plant species (Zaller et al. 2014) and grapevines (Baumgartner et al. 2005). It has also been shown that glufosinate harms soil microbial communities (Pampulha et al. 2007). However, to the best of our knowledge, the current study seems to be among the first showing effects of glufosinate- and flazasulfuron-based herbicides especially on mycorrhizal fungi. These results are important given the wide-reaching effect of AMF on developing systemic resistance in plants protecting them against a wide range of biotrophic and necrotrophic pathogens, nematodes and herbivorous arthropods (Pozo and Azcon-Aguilar 2007; Pineda et al. 2010; Jung et al. 2012; Cameron et al. 2013). Mycorrhizal networks have also been shown to increase the tolerance of grapevines to abiotic stresses (drought, salinity or heavy metals), water stress and biotic stresses such as downy mildew (Trouvelot et al. 2015). Moreover, AMF symbiosis in grapevines affects nutrient uptake and wine quality (Bavaresco et al. 2003) leading to a better oxidative stability and a higher level of bioactive compounds compared to the ones without AMF (Gabriele et al. 2016). In the current study, we only determined overall AMF colonisation, but others found a total of 30 different fungal taxa associated with grapevines including taxa of the genus *Glomus* s.l. and different Glomeromycota taxa (Likar et al. 2013). Moreover, herbaceous weed species have been shown to promote a different set of dominant mycorrhizal fungi, providing a wider spectrum of these fungi for colonizing grapevine roots (Radić et al. 2012), and weed species buffer tillage effects for other soil biota (Buchholz et al. 2017).

Overall, the AMF colonisation rates we found are similar to those reported from Californian vineyards (about 20% averaged over the season; Baumgartner et al. 2005) but are rather low compared with those reported from Slovenian and Croatian vineyards (64–82%, Likar et al. 2013). We think that the low colonisation rates in our study might be due to examining adventive roots emerging directly from the grapevine stem rather than explicitly studying fine-roots.

### Microorganisms in the soil and xylem sap

A growing body of evidence from studies performed under controlled conditions suggests that herbicides can modify soil microbial communities in arable fields or in controlled environments (Wardle and Parkinson 1990; Lo 2010; Zaller et al. 2014; Aristilde et al. 2017). In the current study, soil microbial CFUs varied considerably between herbicides with glufosinate showing the highest, glyphosate the lowest and flazasulfuron medium CFU levels similarly to mechanical control. Only a few other studies examined the influence of herbicides on soil microorganisms in vineyards. Arable field studies under wheat show that glyphosate-herbicide application caused a shift in the species dominating the arbuscular mycorrhizal fungal community in the rhizosphere, possibly through a modified host plant physiology (Sheng et al. 2012). In a study where glufosinate-herbicide significantly increased gram-negative bacterial communities, it was suggested that glufosinate might represent an additional carbon source for the soil bacteria (Kopčáková et al. 2015). Contrastingly, in a rhizobox study following four growth periods, soil bacterial community composition and diversity was less affected by glyphosate-herbicides, although all classes of *Proteobacteria* increased in relative abundance (Newman et al. 2016). Soil bacterial communities in vineyards have been considered as the primary reservoir for most of the bacteria that might colonise the grapevine, affecting at least the first stages of fermentation and perhaps also the wine aroma (Zarraonaindia et al. 2015). A better understanding of the microbial dynamics and their effect on the final product is of great importance to help winemakers produce wine styles of consistency and high quality (Piao et al. 2015).

It is now getting clearer that different viticulture management measures form distinctly different soil microbial communities which affect beneficial interactions with the grapevines (Likar et al. 2017). However, soil microbial communities have been demonstrated to be highly variable depending on viticultural management (e.g. conventional vs. organic), soil management, vineyard age and environmental factors. Hence, it can be expected that interactions between soil type or agricultural practices such as fertilizing or tillage also shape the influence of herbicides on soil biota. Clearly, more research is needed to further study these complex relationships.

Surprisingly, total bacteria counts in xylem sap taken 11 months after herbicide application tended to be on average 170% higher under herbicide-treated plots compared to mechanically weeded grapevine rows. Although this difference was not significantly different due to high variability among samples, it suggests that non-target effects of herbicides cascade through the grapevine system, potentially also affecting chemical and sensory parameters of wine (Piao et al. 2015).

### Earthworms and litter decomposition

The lack of response of earthworm surface casting activity, biomass or density was somewhat surprising, as several studies show detrimental effects of chemical herbicides on earthworms (e.g. Pelosi et al. 2014; Zaller et al. 2014; Gaupp-Berghausen et al. 2015; Travlos et al. 2017). We explain the contrasting effects of the current study as follows: first, we measured earthworm activity by their surface cast production; however, it might well be that earthworms responded by changing their subsurface casting behaviour instead (Zaller et al. 2013). Second, the herbicides were applied within grapevine rows only but not between rows leaving two-thirds of the area untreated. Hence, earthworm could migrate between undisturbed and treated areas or might have avoided herbicide-treated within-rows by going into deeper soil layers or burrowing into neighbouring, untreated areas. Such avoidance behaviour among earthworms was reported earlier (Capowiez et al. 2003; Salvio et al. 2016). Third, these contrasting effects could also be the result of species-specific responses of earthworms (Pelosi et al. 2013). In fact earthworm communities in the current study consisted mainly of *Allolobophora chlorotica*, *A. rosea* and other endogeic species known to be less sensitive to pesticides than species used in the former studies (Pelosi et al. 2013). The lack in earthworm response could also indicate that our study site was already populated by earthworm species that were adapted to year-long pesticide applications (Pauwels et al. 2013; Stellin et al. 2017). This could also explain the lack of effects on cocoon production and hatchling numbers, which contradicted a former study where a 50% reduction in hatchling success (Gaupp-Berghausen et al. 2015) or up to 26% mortality rate (Stellin et al. 2017) after glyphosate-herbicide application was found. Fourth, earthworm sensitivity to pesticides also varies dependently on different doses and herbicide formulations (C Ma and Bodt 1993; Salvio et al. 2016), and age or development stages of earthworms (Lowe and Butt 2007).

Litter decomposition in the current study was unaffected by the employed weed control measures. This lack of a response of litter decomposition is in line with studies investigating effects of glyphosate-herbicides (van Hoesel et al. 2017). Also, soil biological activity measured by biolog-essays did not show herbicide effects (Marwitz et al. 2014). Nevertheless, litter decomposition has been shown to be a sensitive ecotoxicological parameter even being influenced by very small amounts of pesticides used in seed dressings (Zaller et al. 2016). Other reasons for the contrasting results might also be that the current study was performed in the field while those mentioned above have been conducted under controlled conditions or that decomposer communities in our vineyards were already tolerant towards herbicide levels.

### Nutrients in different grapevine compartments

Weed control measures affected nutrient composition in roots, leaves, xylem sap and grape juice. We also found that herbicides had different effects in different grapevine parts (i.e. herbicide × grapevine part interaction). Although the effects were subtle, they demonstrated that weed control can last more than one season and alter grapevine nutrient uptake. A study conducted in Croatian vineyards showed that grapevine leaf concentrations of the macronutrients K and P and the micronutrient Mn were mainly affected by soil K, Fe, Cu, organic matter, grapevine mycorrhization and management system (organic vs. conventional; Likar et al. 2015). The alteration of C and N status after herbicide treatment may affect the grapevine vigour in the long term (Saladin et al. 2003). The negative correlation between grapevine mycorrhization or earthworms and nutrient contents in xylem sap in the current study indicates that there is indeed some relationship between soil biota and grapevine nutrition as shown for other plant species (Trouvé et al. 2014; Zaller et al. 2011a; Likar et al. 2017). In the Champagne region, the herbicide flazasulfuron led to altered growth, yellow leaves and several physiological dysfunctions in grapevines suggesting that the herbicide is actually toxic for grapevines (Magné et al. 2006). Also, other commonly used herbicides in vineyards (fluoroglycofen and acetochlor) caused color changes in grape leaves and growth inhibition in the following year due to an accumulation of starch (Tan et al. 2014). Herbicides used in vineyards (e.g. diuron) are also prone to leaching as shown in an experiment in France (Jacobson et al. 2005).

The current study is among the first showing herbicide-induced changes in nutrient composition of grapevine xylem sap taken 11 months after herbicide application. Especially, sap samples under glyphosate-herbicides showed higher K concentrations than mechanically weeded plots suggesting that glyphosate alleviated K uptake of grapevines. For N, it is known that during the spring, the autumnal N reserves represented 4% of the total pool of nitrogen present in the xylem sap, while 40% of the same pool originated from the spring supply (Glad et al. 1994). More research on the long-term consequences of herbicides on grapevine physiology seems imperative.

## Conclusion

Taken collectively, our findings bring valuable information about herbicide-induced alterations in grapevine mycorrhization, soil microorganisms and nutrient concentration in different grapevine parts. The question arises, whether the benefits of removal of weeds in grapevine rows outweigh potential harm on essential ecological functions within this agroecosystem that could also affect grapevine health or wine quality. The main reason to eradicate weeds is the suspected competition by weeds for water-reduced grape yields. However, surprisingly few studies have proven this relationship (Smith et al. 2008; Ellen Adams 2011; Winter et al. 2018). The current study was a first attempt to further elucidate the wide ramifications of herbicide effects within the vineyard ecosystem. Clearly, more long-term interdisciplinary approaches are needed in the field and/or under controlled conditions when the aim is to develop more ecologically sound viticultural management systems with less herbicide input.

## Electronic supplementary material

Supplementary Table S1 (XLSX 13 kb)
